# Resourcefulness Skills Use by Grandmothers Raising Grandchildren: A Longitudinal Case Study Approach

**DOI:** 10.1093/geroni/igab046.1903

**Published:** 2021-12-17

**Authors:** Alexandra Jeanblanc, Carol Musil, Elizabeth Tracy, Jaclene Zauszniewski

**Affiliations:** 1 Case Western Reserve University, Cleveland, Ohio, United States; 2 CWRU School of Nursing, Cleveland, Ohio, United States

## Abstract

In the U.S., over 2.7 million grandparents are primary caregivers to grandchildren. It is critical to understand the experiences of grandparent caregivers to design tailored, supportive programs. Our aim was to analyze 4 weeks of daily online journals of 129 grandmothers with respect to their use of a set of Resourcefulness Skills© following web-based skills training. Using a thematic analysis approach, coding was completed by a three person team using NVIVO 12. Percent agreement among coders was over 90% (Kappa = .956). Twelve cases were randomly selected for case study development. Comparative case study analysis was used to look within and across cases for instances where skills were used and how skill use changed over time. The pattern of skill use showed that grandmother caregivers used resourcefulness skills to deal with the grandchild’s behavior and developmental issues as well as within the entire family system to manage conflicted relationships with the grandchild’s parents, balance relationships with their spouse/partner, and maintain relationships with other relatives. Case studies will be presented to show skill use over the four weeks of journaling in the context of the family system, as well as the strategies used by participants who improved skill use over time and those who faced barriers to skill use. Findings highlight the use of journals as a means to assess enactment fidelity of treatment interventions and the importance of the family network in skills training program implementation and ways to help grandmothers make use of skills training in the family setting.

